# The efficacy and safety of gabapentin vs. carbamazepine in patients with primary trigeminal neuralgia: A systematic review and meta-analysis

**DOI:** 10.3389/fneur.2023.1045640

**Published:** 2023-05-02

**Authors:** Xin Zhao, Shuyu Ge

**Affiliations:** ^1^Department of Pharmacy, Beilun People's Hospital, Ningbo, China; ^2^Department of Pharmacy, Tongde Hospital of Zhejiang Province, Hangzhou, China

**Keywords:** gabapentin, carbamazepine, primary trigeminal neuralgia, randomized controlled trial, meta-analysis

## Abstract

**Background:**

Drug therapy is the most commonly used treatment for primary trigeminal neuralgia (PTN), in which carbamazepine is the first-line drug. Recently, the anti-epileptic drug gabapentin has also been widely used in patients with PTN, but whether it can be used as a substitute for carbamazepine still needs to be verified. Our study aimed to assess the safety and efficacy of gabapentin vs. carbamazepine as a treatment for PTN.

**Methods:**

We searched seven electronic databases for studies published as of 31 July 2022. All randomized controlled trials (RCTs) of gabapentin vs. carbamazepine on patients with PTN that met the inclusion criteria were included. Meta-analysis was conducted using Revman 5.4 and Stata 14.0, in which forest plots, funnel plots, and sensitivity analysis were performed. Mean difference (MD) and odds ratio (OR) with 95% confidence intervals (CIs) were used for the measurement indicators of continuous and categorical variables, respectively.

**Results:**

A total of 18 RCTs with 1,604 patients were eventually identified. The meta-analysis showed that compared with the carbamazepine group, the gabapentin group significantly improved the effective rate (OR = 2.02, 95% CI 1.56 to 2.62, *P* < 0.001), reduced the adverse event rate (OR = 0.28, 95% CI 0.21 to 0.37, *P* < 0.001), and improved the visual analog scale (VAS) score (MD = −0.46, 95% CI −0.86 to −0.06, *P* = 0.03). Although the funnel plot showed evidence of publication bias, the sensitivity analysis revealed the stability of the results.

**Conclusion:**

The current evidence showed that gabapentin may be superior to carbamazepine in relation to efficacy and safety in patients with PTN. It is crucial that more RCTs are conducted to confirm the conclusion in the future.

## Introduction

Trigeminal neuralgia (TN) is a common peripheral neuropathy in neurology. The characteristic symptoms of TN include unilateral, paroxysmal, provocable, and without sensory loss of facial pain (1). It can be divided into primary trigeminal neuralgia (PTN, i.e., idiopathic and classical types of TN) and secondary trigeminal neuralgia (STN), of which PTN is more common (2–4). Various examinations revealed that PTN refers to pain with clinical symptoms, while without a clear etiology, and no organic lesions related to the pathogenesis (5). STN refers to pain caused by a clear etiology, such as intracranial tumors, inflammation, and abnormal blood vessels (6).

There are more women with PTN, and it is more common after the age of 40. It can be described by patients as electrocautery, tearing, acupuncture, or knife cutting. At the onset, patients often show a painful expression, and it is necessary to rub the face tightly with a towel to relieve the pain. The onset of the disease is acute, and it stops suddenly after 1–2 min and relapses after a period of time. The interval between relapses shortens as the disease aggravates (7). At present, the etiology, lesion location, and pathogenesis of PTN are not completely clear. There are clinical theories of central etiology and mechanical compression, as well as the theory of microvascular, which need further research to confirm (8).

At present, drugs can be used for nerve block, and then, neurotomy, microvascular decompression, or radiofrequency thermocoagulation can be used for treatment, but available treatments are still far from the ideal effect and pain tend to recur (9–12). Carbamazepine is the first choice for the treatment of primary trigeminal neuralgia, and it is effective for most patients, but it has many adverse reactions; blood routine, electrolytes, and liver and kidney functions should be monitored during medication (13).

Gabapentin has been used in clinics for more than 10 years as a new type of anti-epileptic drug. Its good curative effect for neuropathic pain has been reported in many literature studies (14, 15). PTN is also a neuropathic pain in mechanisms. At present, many randomized controlled trials (RCTs) comparing the safety and efficacy of gabapentin and carbamazepine for PTN have been carried out. Whether gabapentin can be used as a substitute for carbamazepine has not been verified by evidence-based research. Our study conducted a systematic review and meta-analysis of RCTs of gabapentin and carbamazepine as a treatment for PTN, so as to provide evidence-based medicine for medical practice.

## Methods

### Literature search strategy

Seven electronic databases (PubMed, Web of Science, EMBASE, Chinese BioMedical Database, China National Knowledge Infrastructure, China Scientific Journal Database, and Wanfang Database) were comprehensively searched from inception to 15 August 2022 for all studies involving gabapentin vs. carbamazepine for the treatment of PTN. The combination of “gabapentin”, “carbamazepine”, and “primary trigeminal neuralgia” used the Boolean operator “AND” to carry out the specific search. Language restrictions and publication status were not considered in our literature search. A hand search of the reference lists of the original articles and previous reviews was also conducted to screen other potentially eligible studies.

### Study selection

The inclusion criteria were as follows: (P) patients with primary trigeminal neuralgia (PTN); (I) intervention: the test group received oral gabapentin treatment; (C) comparison: the control group received oral carbamazepine treatment; (O) main outcome indicators: the effective rate, the adverse event rate, and the visual analog scale (VAS) score; and (S) study design: randomized controlled trials (RCTs). The major exclusion criteria were as follows: (1) patients with secondary trigeminal neuralgia (STN) or other diseases; (2) non-randomized controlled studies; (3) treatment with other interventions; and (4) the relevant data not reported.

### Data extraction

XZ and SG independently reviewed RCTs included in the final analysis and extracted relevant data directly from the articles. The following information was extracted from each included study: author's name, year of publication, treatment of test and control group, sample size, patients' characteristics, study's years of onset, and the results of main outcome indicators [the effective rate, the visual analog scale (VAS) score, and the adverse event rate].

The VAS score was used to assess the pain degree of patients, with a total score of 10 points. In order to categorize the patients, the VAS score was divided into three categories: mild or no pain (0–2), moderate pain (3–6), and severe pain (7–10). Compared with before treatment, the VAS score decreased by >50% after treatment indicated effective treatment; otherwise, it was considered ineffective.

### Quality assessment

Quality assessment of RCTs was performed using the Cochrane bias risk assessment tool (The Cochrane Collaboration) by the following items: random sequence generation, allocation concealment, blinding (performance bias and detection bias), incomplete outcome data (attrition bias), selective reporting (reporting bias), and other bias.

### Statistical analysis

All statistical analyses were performed by using Review Manager, version 5.4, software (The Nordic Cochrane Centre) and Stata 14.0 (STATA Corp., College Station). Specifically, data for effect sizes of continuous outcomes were calculated as mean difference (MD) with a 95% confidence interval (CI), and odds ratio (OR) was used for categorical outcomes. Heterogeneity between studies was assessed using the I^2^ statistic the Cochrane Q-test. Statistical heterogeneity will determine the choice of model (fixed or random effect). An assessment of possible publication bias was carried out using funnel plots and Egger's test. If I^2^ is >50% or the *p*-value of publication bias is < 0.05, the potential source of heterogeneity or publication bias would be tested by sensitivity analysis, which was evaluated by removing study after study from the pooled results using the leave-one-out method.

## Results

### Search process

**Figure 1:**
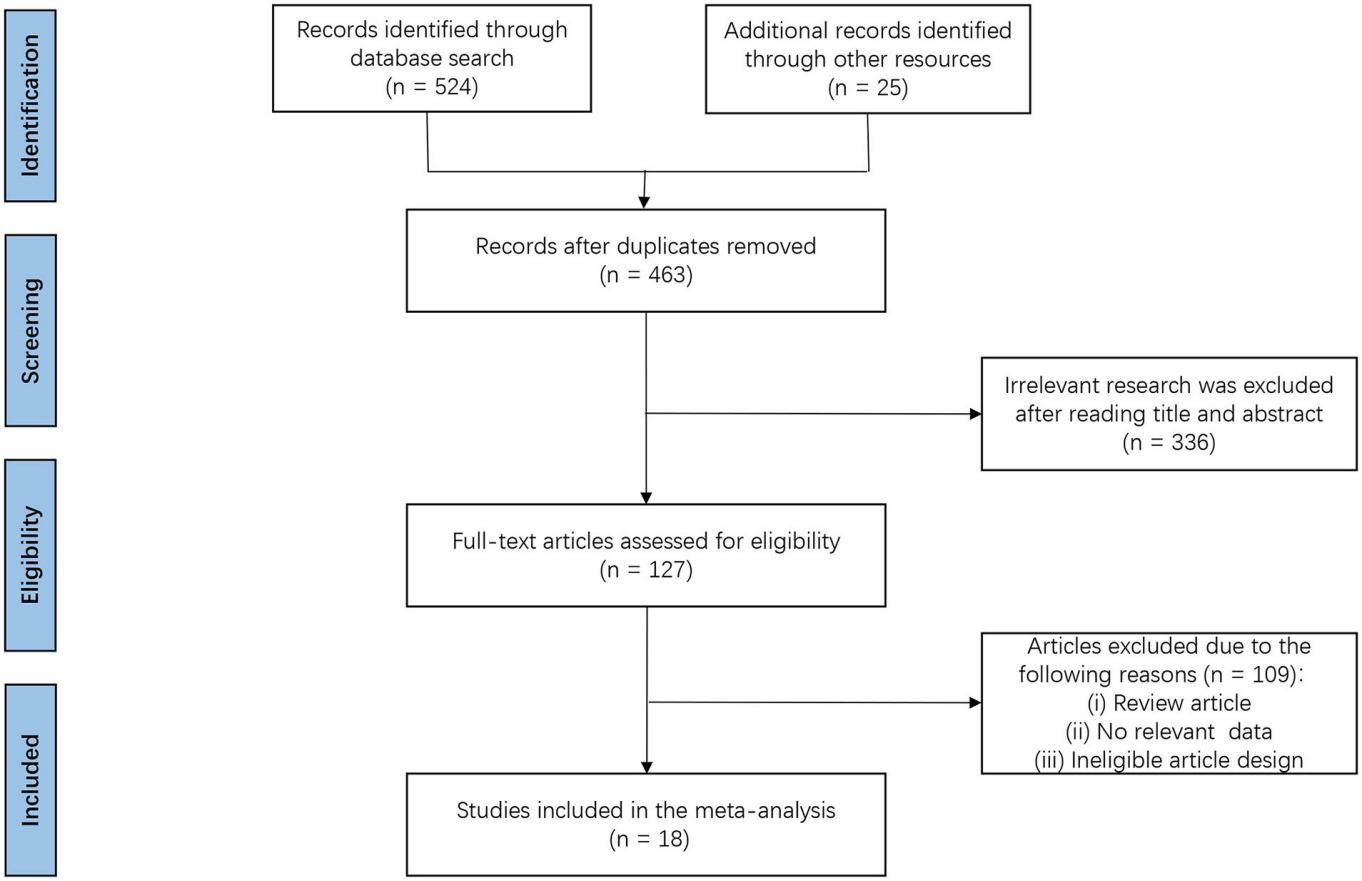
Flow diagram of literature search and study selection.

A total of 549 potentially relevant articles were identified by the searches. After the removal of duplicates, 463 articles were identified. After going through the titles and abstracts, another 336 articles were excluded. Full-text articles from the remaining 127 studies were assessed for eligibility, and 109 studies were further excluded because they did not meet the inclusion criteria. Eventually, 18 RCTs were deemed eligible and were included in the final meta-analysis (16–33). Figure 1 illustrates the search process and application of the study inclusion/exclusion criteria.

### Characteristics of the included studies

The detailed characteristics of these 18 eligible studies are summarized in Table 1. This study included 18 RCTs consisting of 1,604 cases, of which 802 were in the test group and 802 were in the control group. The sample size was between 40 and 210, the treatment time ranged from 28 days to 60 days, and the studies were published between 2008 and 2020.

**Table 1:** Presentation of main characteristics for included studies.

**Study**	**Treatment**		**Treatment time (days)**	**No. of patients**		**Gender (M/F)**		**Age (years)**		**Course of disease (months)**		**Years of onset**
	**Test**	**Control**		**Test**	**Control**	**Test**	**Control**	**Test**	**Control**	**Test**	**Control**	
Chen (16)	GBP: 200–1300 mg, tid	CBZ: 200–500 mg, tid	28	49	49	27/22	29/20	53.8 ± 11.6	52.1 ± 11.3	NR	NR	March 2015 to March 2017
Fan et al. (23)	GBP: 100–800 mg, tid	CBZ: 100–300 mg, tid	60	31	31	18/13	19/12	52.21 ± 6.58	53.01 ± 6.21	20.14 ± 5.23	19.61 ± 6.31	February 2013 to February 2014
Gu et al. (31)	GBP: 100–800 mg, tid	CBZ: 200–300 mg, tid	70	34	34	16/18	15/19	52.13 ± 5.75	53.57 ± 5.14	NR	NR	September 2007 to April 2009
Huang et al. (28)	GBP: 300–600 mg, tid	CBZ: 100–300 mg, tid	28	48	48	22/26	23/25	56.13 ± 3.75	55.73 ± 4.97	NR	NR	February 2009 to June 2011
Li (21)	GBP: 300–1200 mg, qd	CBZ: 100–200 mg, bid	30	45	45	14/31	16/29	57.13 ± 1.06	56.09 ± 1.12	9.56 ± 1.03	9.96 ± 1.12	January 2014 to January 2015
Liu (25)	GBP: 300–1200 mg, tid	CBZ: 100–400 mg, tid	28	36	36	16/20	15/21	66.18 ± 4.92	67.01 ± 5.28	NR	NR	May 2017 to October 2018
Liu (33)	GBP: 300–800 mg, tid	CBZ: 100–400 mg, tid	28	40	40	18/22	16/24	49.2 ± 4.3	49.2 ± 4.7	NR	NR	January 2018 to December 2019
San et al. (30)	GBP: 300–600 mg, tid	CBZ: 100–300 mg, tid	35	20	20	11/9	8/12	30–77	32–75	NR	NR	April 2015 to April 2016
Wang (27)	GBP: 300–600 mg, tid	CBZ: 100–600 mg, tid	28	105	105	55/50	51/54	55.65 ± 2.59	56.02 ± 2.66	NR	NR	March 2015 to March 2019
Wang (18)	GBP: 300–800 mg, tid	CBZ: 100–300 mg, tid	60	36	36	22/14	23/13	53.4 ± 11.6	53.5 ± 11.8	22.6 ± 9.8	22.4 ± 9.6	August 2011 to August 2012
Wang (22)	GBP: 300–1200 mg, tid	CBZ: 150–600 mg, tid	28	44	44	30/14	29/15	52.21 ± 6.58	53.01 ± 6.21	20.14 ± 5.23	19.62 ± 6.31	June 2017 to December 2018
Yang (19)	GBP: 100–300 mg, tid	CBZ: 100–300 mg, tid	60	42	42	19/23	20/22	54.2 ± 5.5	55.4 ± 5.6	10.44 ± 2.58	10.45 ± 2.59	August 2013 to May 2015
Zhang and Xiao (26)	GBP: 300–1200 mg, tid	CBZ: 100–400 mg, tid	60	40	40	11/29	14/26	56.2 ± 5.1	56.7 ± 5.2	10.1 ± 2.4	10.2 ± 2.5	January 2017 to June 2018
Zhao (24)	GBP: 300–600 mg, tid	CBZ: 100–300 mg, tid	28	49	49	31/18	28/21	45 ± 1.29	44 ± 1.72	NR	NR	February 2015 to February 2016
Zhou et al. (32)	GBP: 300–1200 mg, tid	CBZ: 100–400 mg, tid	28	44	44	17/27	19/25	51.9 ± 7.2	52.6 ± 7.8	12.1 ± 6.5	11.5 ± 6.3	February 2014 to December 2015
Zhou (20)	GBP: 300–800 mg, tid	CBZ: 100–500 mg, tid	30	51	50	23/28	24/26	44 ± 1.7	45 ± 1.5	10.51 ± 2.63	10.24 ± 2.67	January 2012 to January 2014
Zhou (29)	GBP: 300–1200 mg, tid	CBZ: 100–400 mg, tid	28	43	43	20/23	18/25	56.12 ± 4.69	56.45 ± 4.71	10.51 ± 2.63	10.43 ± 2.57	September 2011 to September 2012
Zhu et al. (17)	GBP: 300–1200 mg, tid	CBZ: 100–400 mg, tid	28	45	46	23/22	22/24	61.03 ± 8.30	59.68 ± 8.72	5.57 ± 1.02	5.43 ± 0.91	January 2006 to June 2007

GBP, gabapentin; CBZ, carbamazepine; qd, one time daily; bid, twice daily; tid, three times daily; NR, not reported.

### Results of quality assessment

**Figure 2:**
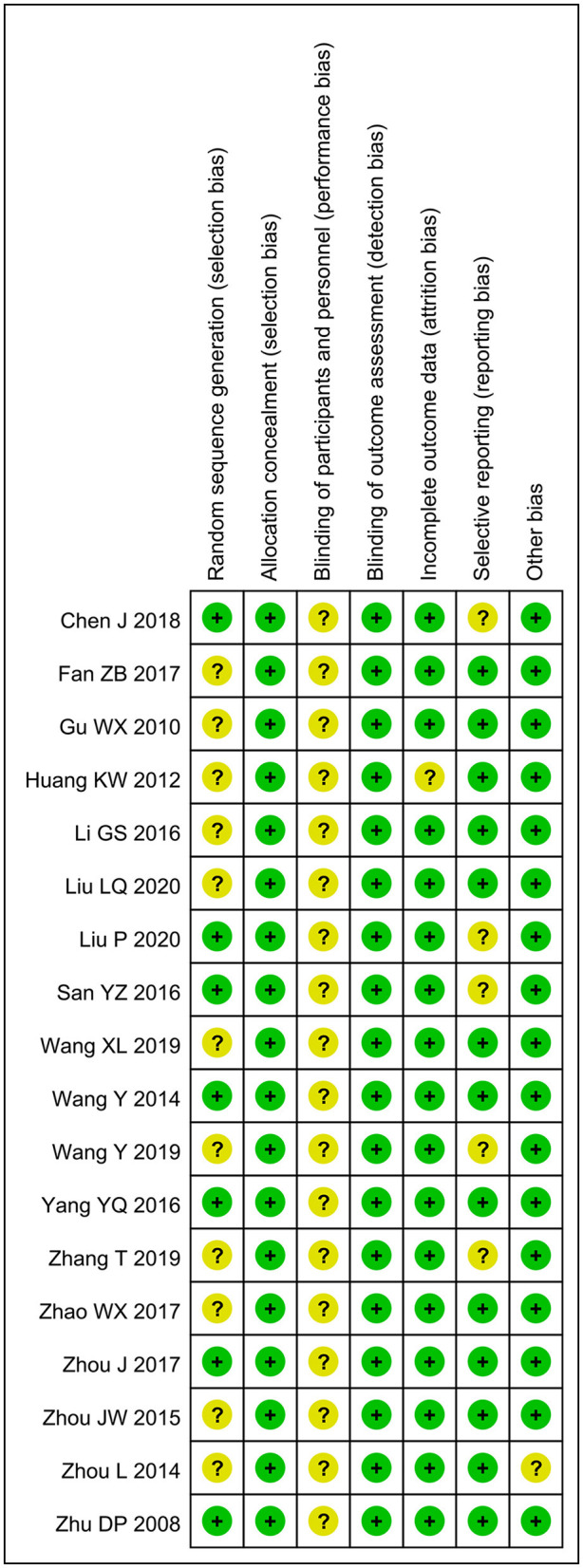
Risk of bias summary: review authors' judgments about each risk of bias item for each included study: low (green), unclear (yellow), and high (red).

The included RCTs were assessed for bias using the Cochrane Risk of Bias Assessment tool (Figure 2). Among them, 11 articles had selection bias as they did not use the random number table for grouping, all articles did not mention the use of blinding, one article had cases lost to follow-up and had attrition bias, five articles had reported bias due to fewer outcome variables, and another article had other biases due to the lack of comparability between the two groups. Overall, none of the included RCTs show a high risk of bias, and their quality was acceptable.

### Results of meta-analysis

#### Effective rate

**Figure 3:**
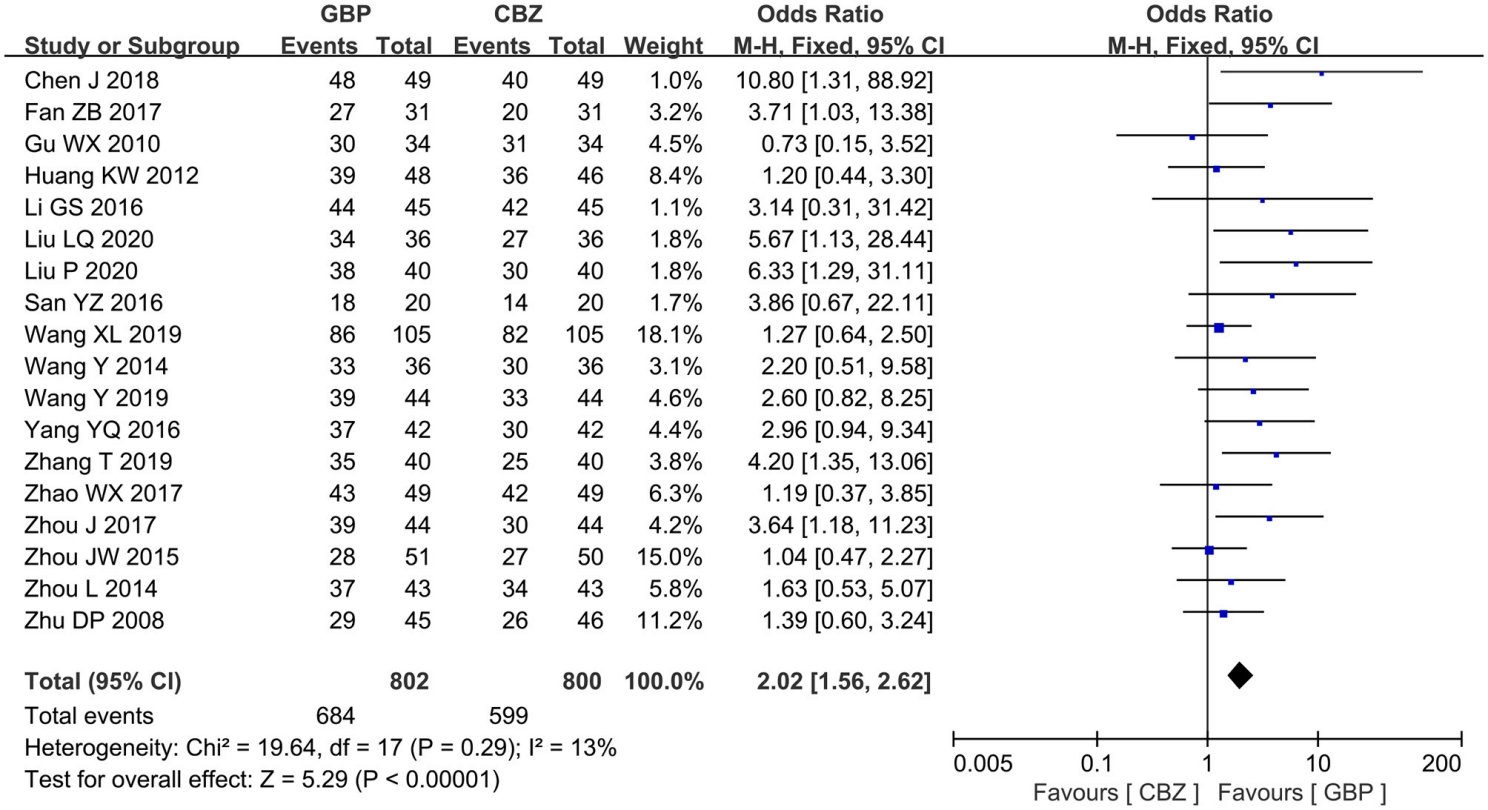
Forest plot of GBP vs. CBZ: effective rate. GBP, gabapentin; CBZ, carbamazepine.

All the included studies measured the effective rate after treatment. The included studies showed no notable heterogeneity (I^2^ = 13%, *P* = 0.29), so a fixed-effects model was selected for analysis, which revealed that the gabapentin group showed a high effective rate compared with the carbamazepine group after treatment (OR = 2.02, 95% CI: 1.56 to 2.62, *P* < 0.00001) (Figure 3).

#### VAS score

**Figure 4:**
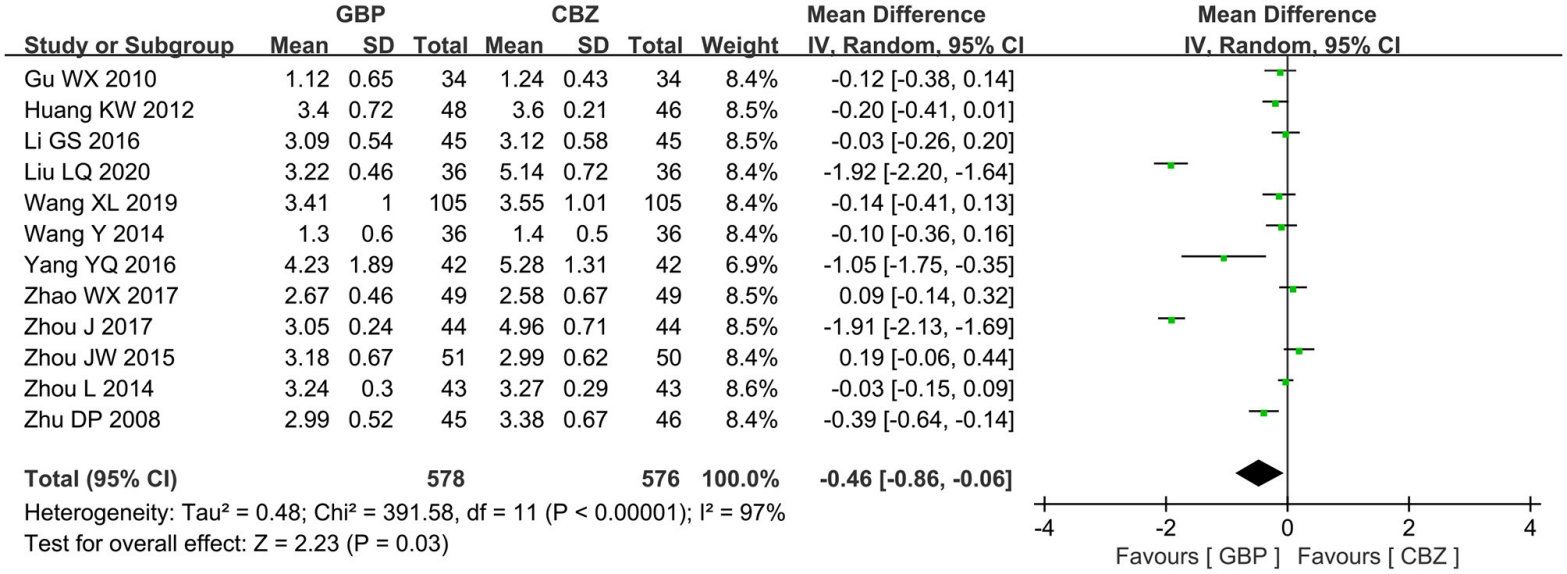
Forest plot of GBP vs. CBZ: VAS score. GBP, gabapentin; CBZ, carbamazepine.

A total of 12 articles reported the VAS score of gabapentin vs. carbamazepine in treating patients with PTN. The included studies had marked heterogeneity (I^2^ = 97%, *P* < 0.00001), so the random effects model was selected for further analysis, and it showed that the gabapentin group had a lower VAS score than the carbamazepine group (MD = −0.46, 95% CI: −0.86 to −0.06, *P* = 0.03) (Figure 4).

#### Adverse event rate

**Figure 5:**
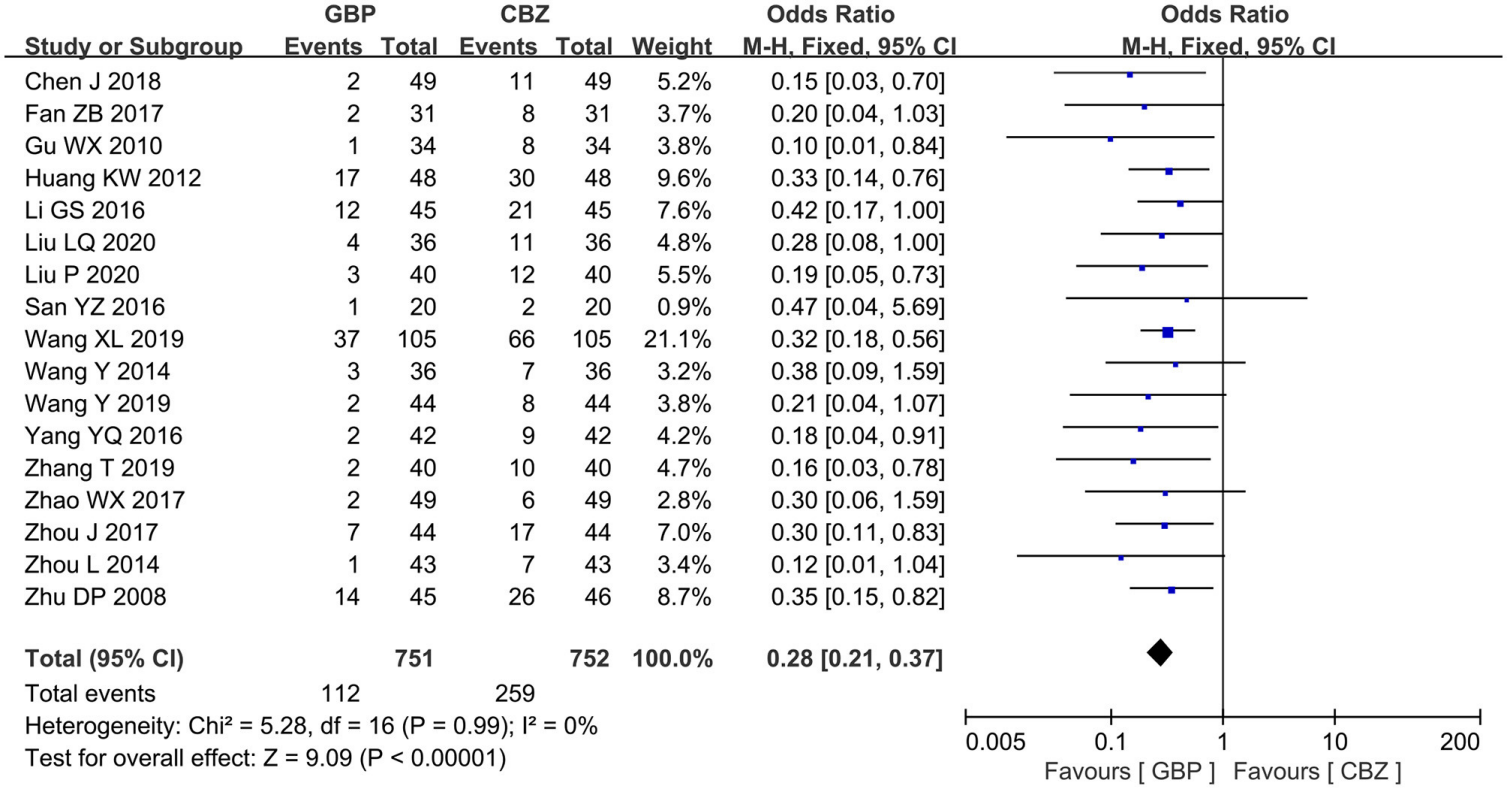
Forest plot of GBP vs. CBZ: adverse event rate. GBP, gabapentin; CBZ, carbamazepine.

A total of 17 studies had data available to assess the adverse event rate after treatment. No heterogeneity was found among studies (I^2^ = 0%, *P* = 0.99). A significant decrease in adverse event rate was observed in the gabapentin group when compared with the carbamazepine group (OR = 0.28, 95% CI: 0.21 to 0.37, *P* < 0.00001) (Figure 5).

#### Publication bias

**Figure 6:**
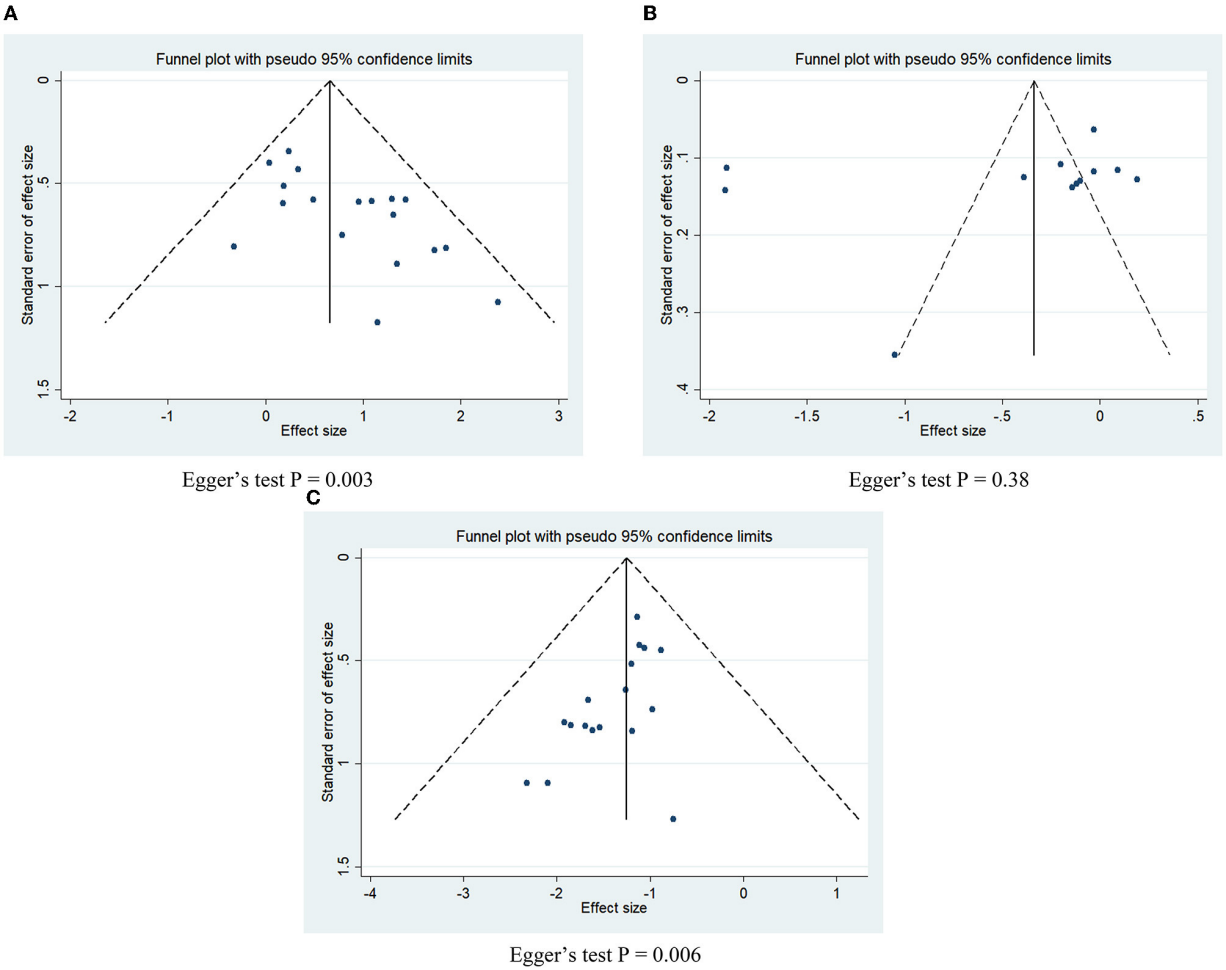
Funnel plots and Egger's test for potential publication bias. **(A)** Effective rate; **(B)** VAS score; **(C)** adverse event rate.

The funnel plots corresponding to the three outcome variables were visually asymmetric (Figure 6), and the *p*-values of Egger's test for the effective rate and the adverse event rate were <0.05 (*P* = 0.003 for the effective rate, Figure 6A; *P* = 0.006 for the adverse event rate, Figure 6C), indicating that there may be potential publication bias between the two variables included in the literature; the *p*-value of Egger's test for VAS score was 0.38 (Figure 6B), indicating that no obvious publication bias was present.

#### Sensitivity analysis

**Figure 7:**
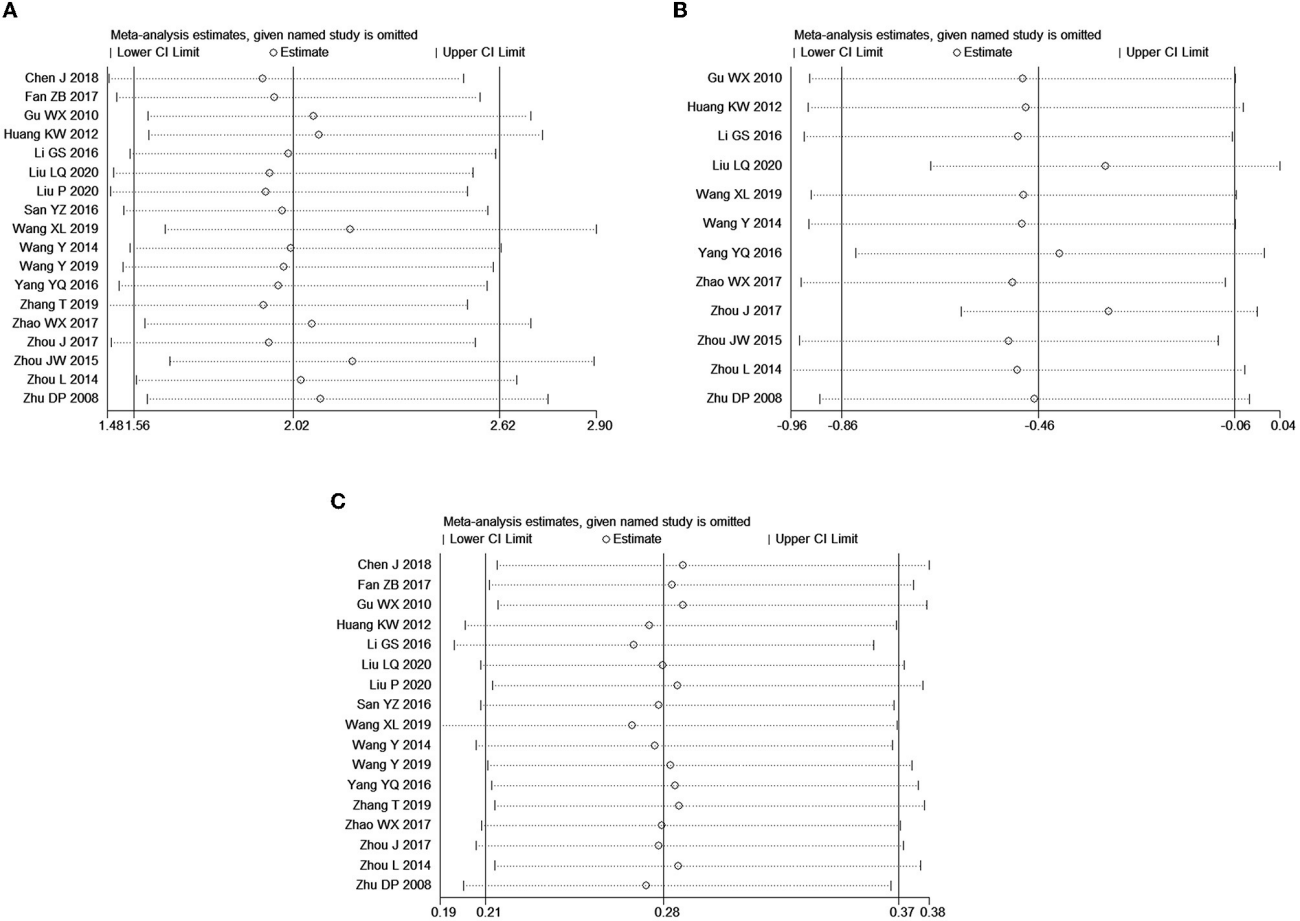
Sensitivity analysis of meta-analysis for outcomes. **(A)** Effective rate; **(B)** VAS score; **(C)** adverse event rate.

Since there was publication bias among the included literature on the effective rate and adverse reaction rate (*P* < 0.05), and significant heterogeneity existed in the included literature for VAS score, we conducted a sensitivity analysis on the meta-analysis results of the three variables (Figure 7). Arbitrary deletion of an included article did not change the significance of meta-analysis results for the effective rate (Figure 7A) and adverse event rate (Figure 7C), which indicated that the meta-analysis results of these two variables were stable, while after deletion of Liu (25), the combined results of VAS scores (Figure 7B) become statistically insignificant, which indicated that the meta-analysis of VAS was less stable.

## Discussion

An episode of PTN is often triggered by normal facial activities such as speaking, chewing, brushing, and washing, or by touching an area of the mouth or face called a “trigger point” (34). In order to avoid attacks, the patient should try to reduce the actions such as speaking, chewing, brushing their teeth, and washing their face, so as to avoid physical and psychological suffering. At present, the etiology of TN is not completely clear. The classic theory holds that the occurrence of TN is due to the compression of abnormally twisted blood vessels such as the superior cerebellar artery and basilar artery near the pons of the trigeminal nerve root. The nerve root is compressed by an aneurysm or arteriovenous malformation (8). Studies have shown that vascular compression at the root of the trigeminal nerve can be found in 95% of patients with PTN (35).

In the present meta-analysis, we comprehensively reviewed and pooled results from 18 RCTs with a total of 1,604 patients who compared gabapentin and carbamazepine therapy for PTN. The results revealed that for patients with PTN, the gabapentin group resulted in a significant improvement in the effective rate compared with the carbamazepine group with an OR of 2.02 and a significant decrease in adverse event rate with an OR of 0.28. The pooled result of the VAS score in the gabapentin group was significantly lower than the carbamazepine group.

There was a meta-analysis to evaluate the safety and efficacy of gabapentin for diabetic peripheral neuralgia. The results showed that gabapentin has a significant effect in the treatment of diabetic peripheral neuralgia with good safety, and its efficacy was comparable to that of carbamazepine or oxcarbazepine. The effective rate was even lower than that of carbamazepine or oxcarbazepine (36). There were also studies on the safety and efficacy of gabapentin for post-herpetic neuralgia (37, 38). Evidence showed that gabapentin was effective for post-herpetic neuralgia and had a high treatment retention rate. These studies confirmed that gabapentin was effective in treating a wide variety of neuropathic pain symptoms. María's study conducted a systematic review of the efficacy of pregabalin and gabapentin for pain and disability due to acute sciatica and adverse events associated with their clinical use and found that gabapentin compared with placebo in assessing leg pain, low back pain, and function. There was no statistical difference in the remaining period of disability, suggesting that gabapentin may be less effective in acute neuropathic pain (39).

The chemical name of gabapentin is 1-(aminomethyl)-cyclohexaneacetic acid, and its molecular structure is related to the neurotransmitter γ-aminobutyric acid. It has been widely used for TN in recent years, but its analgesic mechanism has not been fully elucidated. Its molecular structure is similar to that of γ-aminobutyric acid, and it is a voltage-gated calcium channel c281 subunit blocker (40, 41). At present, it is believed that it can act on peripheral nerve nociceptors, spinal pain conduction pathway, cerebral cortex, and other targets, regulate voltage-gated calcium channel receptors, and reduce the release of excitatory neurotransmitters to achieve the analgesic effect. Therefore, gabapentin may have a therapeutic effect on PTN (42).

Studies have found that inflammatory factors are closely related to the occurrence and progression of TN. Due to changes in nerve demyelination, intraneural mast cells, macrophages, and vascular endothelial cells are damaged and inflammatory responses are induced (43). Tumor necrosis factor-α (TNF-α) can promote the aggregation and activation of inflammatory cells and induce neuropathic pain. Interleukin-6 (IL-6) has a variety of cellular functions and has the effect of promoting inflammation and inducing the production of acute response protein (44). Gabapentin has been confirmed to reduce the levels of TNF-α and IL-6 and inhibit the inflammatory response of patients, thereby achieving the effect of relieving pain and alleviating clinical symptoms (33).

This study had the following limitations: (1) The allocation sequence concealment and risk of blinding bias in most studies were unclear, and there may be limitations; (2) although all included studies had the same purpose, each included study differed in terms of patient disease severity and drug dosage. There were different degrees of differences in terms of time and intervention time, and these differences may lead to heterogeneity of outcomes. (3) The currently included studies were all published in Chinese, which may affect the extrapolation of the results of this study. Therefore, the final conclusion is still needed to be further verified by more large-sample, multi-center high-quality RCTs.

## Conclusion

Existing evidence shows that gabapentin is effective in the treatment of PTN, and it is even better than carbamazepine in terms of therapeutic effect and safety. Gabapentin may be a better choice in cases where first-line drugs such as carbamazepine are ineffective in controlling pain or have severe side effects. In addition, the findings of this review are limited by the quality and limitation of the included studies, and more high-quality studies subdivided research should be further carried out.

## Data availability statement

The raw data supporting the conclusions of this article will be made available by the authors, without undue reservation.

## Author contributions

SG designed the study. XZ performed a database search, data extraction, and quality evaluation. SG and XZ performed the statistical analysis, verified the data, and drafted the manuscript. All authors contributed to the article and approved the submitted version.
